# Cochrane corner: community-level interventions to increase access to food in low- and middle-income countries

**DOI:** 10.11604/pamj.2022.43.163.35415

**Published:** 2022-12-01

**Authors:** Solange Durão, Vundli Ramokolo, Anel Schoonees, Scott Drimie

**Affiliations:** 1 Cochrane South Africa, South African Medical Research Council, Francie Van Zijl Dr, Parow Valley, Cape Town, 7501, South Africa,; 2 HIV Prevention Research Unit, South African Medical Research Council, Francie Van Zijl Dr, Parow Valley, Cape Town, 7501, South Africa,; 3 Centre for Evidence-based Health Care, Division of Epidemiology and Biostatistics, Faculty of Medicine and Health Sciences, Stellenbosch University, Francie van Zijl drive, Parow Valley, Cape Town, 7501, South Africa,; 4 Faculty of Medicine and Health Sciences, Stellenbosch University, Francie van Zijl drive, Parow Valley, Cape Town, 7501, South Africa

**Keywords:** Food security, food access, cash transfers

## Abstract

Food insecurity and hunger is a continuing problem in Africa, with access to food being critical to address it. Community-level interventions may help to increase access to food, specifically, nutritious food. We highlight a Cochrane review that assessed community level interventions aiming to increase access to nutritious food in low- and middle-income countries (LMICs), including those that improve buying power, address food prices, and the social environment. Randomised controlled trials and prospective controlled studies that assessed the effects of these interventions on food security and nutritional status were included, providing relevant implications for practice in LMICs. Findings suggest that unconditional cash transfers (UCTs) are important for improving food security, and that UCTs and food vouchers may increase dietary diversity and reduce stunting. This highlights the importance of current programmes in Africa, the need to reflect and refine where needed, and expand their capacity. A holistic approach to address food insecurity in the region.

## Commentary

In this commentary we discuss the findings of a Cochrane review on the effects of community-level interventions for improving access to food in low- and middle-income countries (LMICs) [1]. We focus the article on the implications of the findings for the African context. Globally, the prevalence of undernourishment is no longer declining, and has increased from 8.4 to 9.9 percent in one year, largely due to the influence of COVID-19 pandemic and its mitigation strategies, such as national lockdowns [2]. More than one third of the world´s undernourished population is in Africa, with hunger affecting 21% of its populace [2]. Food security depends, in part, on having physical and economic access to food; community-level interventions may help to increase access to nutritious food.

The Cochrane review assessed the effects of community-level interventions that aimed to improve access to nutritious food in LMICs, for both the whole community and for disadvantaged or at-risk individuals or groups within a community, on food security and nutritional status outcomes. Randomised controlled trials (RCTs) and prospective controlled studies of adults and children living in communities in LMICs were included. Eligible interventions were broadly categorised into those that: i) improve buying power (e.g. create income-generation opportunities, cash transfer schemes); ii) address food prices (e.g. vouchers and subsidies); iii) address infrastructure and transport that affected physical access to food outlets; and iv) address the social environment and social support (e.g. from family, neighbours or government).

A comprehensive search was conducted in 16 databases in September 2019 and updated in six key databases in February 2020 to identify eligible studies. The authors followed Cochrane methodology for screening, data extraction and analysis. The risk of bias of the included studies was assessed using the Effective Practice and Organization of Care (EPOC) risk of bias tool for studies with a separate control group [3]. Random-effects meta-analyses were carried out if there were at least two sufficiently homogeneous studies in the same intervention category reporting a common outcome measure. Where meta-analysis was not possible, the evidence was synthesised using vote counting based on effect direction [4]. The certainty of the evidence was assessed and interpreted using the Grading of Recommendations, Assessment, Development and Evaluation (GRADE) approach [5,6].

After screening 15,477 deduplicated records, 59 studies were included that assessed six different types of interventions (Table 1). Most of the included studies assessed interventions aiming to improve buying power (n=52/59), and particularly addressed unconditional cash transfers within this intervention category (n=21/52). We found that *unconditional cash transfers* (UCTs) improve food security but make little or no difference to child cognitive function and development (high-certainty evidence). Furthermore, they may increase dietary diversity and reduce stunting (low-certainty evidence). The evidence of their effects on the proportion of household expenditure on food and on wasting had very low certainty. *Conditional cash transfers* (CCTs) were found to result in little to no difference in the proportion of household expenditure on food, and to slightly improve cognitive function in children (high-certainty evidence). *Conditional cash transfers* probably slightly improve dietary diversity (moderate-certainty evidence) but may make little to no difference to stunting or wasting (low-certainty evidence). Two prospective controlled studies reported that CCTs make no difference to the proportion of overweight children. *Income generation interventions* were found to probably make little or no difference to stunting or wasting (moderate-certainty evidence). They may result in little to no difference to food security (low-certainty evidence) and may improve dietary diversity in children but not for households (low-certainty evidence). *Food vouchers* were found to probably reduce stunting (moderate-certainty evidence). They may improve dietary diversity slightly (low-certainty evidence) and may result in little to no difference in wasting (low-certainty evidence). *Food and nutrition subsidies* may improve dietary diversity among school children (low-certainty evidence) but the evidence was very uncertain about the effects on household expenditure on healthy foods as a proportion of total expenditure on food (very low-certainty evidence). *Social support interventions* such as community grants probably make little or no difference to wasting (moderate-certainty evidence) and they may make little or no difference to stunting (low-certainty evidence). The evidence of the effects of village savings and loans on food security and dietary diversity was of very low certainty.

**Table 1:** summary of interventions and study designs included

Intervention category	Intervention types	Number of studies (study design)
**Improve buying power**	Unconditional cash transfers	18 RCTs; 3 PCS
	Conditional cash transfers	9 RCTs; 5 PCS
	Income generation	6 RCTs; 11 PCS
**Food prices**	Food vouchers	4 RCTs; 0 PCS
	Food rebates/subsidies	1 RCT; 3 PCS
**Infrastructure/transport**	n/a	0 RCTs; 0 PCS
**Social environment**	Village savings and loans	1 RCT; 1 PCS

RCT: randomised controlled trials; PCS: prospective controlled studies; NOTE: some studies assessed more than one intervention type and are thus duplicated in the table

## Conclusion

The evidence indicates that UCTs can improve food security status and that income-generation interventions do not seem to improve food security. The evidence regarding the effects of the other investigated interventions on food security is unclear. *Conditional cash transfers*, UCTs, income-generation interventions, and food and nutrition vouchers and subsidies can potentially improve dietary diversity. Unconditional cash transfers and food vouchers may reduce stunting, but CCTs, income-generation or social environment interventions do not make a difference on wasting or stunting. *Conditional cash transfers* seem to positively impact cognitive function and development but not UCTs, which may be related to the conditionalities of CCTs, e.g. attending school or regular clinic visits.

The evidence regarding UCTs has important implications; they were found to improve food security and they may improve dietary diversity and reduce stunting. Social transfers in the form of grants, both conditional and unconditional, enhance access to food, but do not necessarily translate into improvements in nutrition status. All countries in sub-Saharan Africa (SSA) have cash transfer programmes in some form and these have been expanding in the region [7]. One type of UCT is the Child Support Grant (CSG), which for example exists in Kenya and South Africa. In South Africa, the grant is not sufficient to cover basic food needs with households often pooling their income to cover the food and non-food needs (e.g. taxi fare, electricity or mobile data) of all members, not only beneficiaries [8]. Apart from affecting food access, income poverty compromises access to water, sanitation and health care services giving rise to frequent infections that further undermine nutritional status. Social grants thus have multiple uses and multiple users. Yet the up-take of the CSG in South Africa remains low - especially in the first few months of life - with one in three infants in poor households not benefiting from social assistance [8]. In sub-Saharan Africa overall, the coverage of cash transfer programmes is low, with a lot of vulnerable households not benefiting, though there are wide disparities in coverage across the region [7]. These gaps in social assistance for very young children are important as they are most vulnerable to both the immediate shock and long-term effects of malnutrition. Expansion of these programmes is important to increase their value and deepen impact. Specifically, the value of the transfers should be adequate to cover basic needs, i.e. to be above the food poverty line and index-linked to the cost of a nutritious food basket to retain their purchasing power. Based on data from 18 SSA countries, the transfer values covered from 7% to over 140% of monthly per capita Gross domestic product (GDP) [7].

Food vouchers possibly reduce stunting and may improve dietary diversity slightly. Placing food vouchers into the hands of the most at-risk individuals in targeted communities is important in this regard. This also highlights the potential of food vouchers and other interventions, including UCTs, CCTs and income-generation interventions, that aims to help minimise the impact of food prices to potentially improve dietary diversity. This points to a more holistic approach to align these with other interventions or conditions to tackle the high levels of child malnutrition.

*Conditional cash transfers* seem to positively impact cognitive function and development which may be related to the conditionalities such as attending school or regular clinic visits. The long-term effects of chronic child malnutrition are severe for individuals, communities and society as a whole. Thus, it is essential that this receives the attention it deserves. The COVID-19 pandemic has showed us that the public and private sectors can rapidly work together to generate funds and implement interventions in response to a threat to the lives and livelihoods of individuals and communities. Chronic child malnutrition remains a global problem despite investments in research and interventions, and awareness campaigns through all forms of media.
